# Supplementary material for: Impact of peripheral artery disease on short-term outcomes after percutaneous coronary intervention: A report from Japanese nationwide registry
Source: PLoS One. 2020 Oct 6;15(10):e0240095. doi: 10.1371/journal.pone.0240095 (PMC7537874; doi:10.1371/journal.pone.0240095)
Supplement: Attachment — Submitted filename: PONE-D-20-00744.pdf [file pone.0240095.s001.pdf]

# PONE-D-20-00744

## Impact of peripheral artery disease on short-term outcomes after percutaneous coronary intervention: A report from Japanese nationwide registry

The authors investigated the impact of peripheral artery disease on in-hospital mortality using a large nationwide registry. Results from their analysis (table 4) show that peripheral artery disease is independently associated with in-hospital mortality.

The authors have access to a huge dataset $n = 894,014$ with many variables and outcomes. Since the number of observations is huge, it is possible to fit complex models to the data. One can, for instance, explore non-linear effects or many interactions between risk-factors. Based on table 4, however, a simple additive model with linear effects was fitted to the data. In addition, continuous risk-factors (e.g. blood-pressure) were dichotomized. I believe it is better to leave the continuous variables untouched as dichotomization leads to all kinds of problems (e.g. assumption of sudden effect, information loss, use of arbitrary cut-offs)[1].

I suggest the authors do not categorize the risk-factors and explore more complex models with non-linear effects.

- Page 2 line 11: What does the number after the +- symbol mean? Please explain in the manuscript.
- Page 2 line 11: Are these the results from the multi-variable model? Please mention in the manuscript.
- Pages 27 and 28 tables 3 and 4: Are some of the hospital outcomes not predictive for mortality? If a risk-factor as 'Acute heart failure within 24 hours' is added to the model, why not 'Cardiogenic shock'?
- All tables: Why report p-values with this number of observations. P-values are not really relevant.
- Page 28 table 4: Please change the unit of age (e.g. per 10 years). The effect OR: 1.04 (95%CI: 1.04-1.04) is not really informative.

[1] https://onlinelibrary.wiley.com/doi/abs/10.1002/0470011815.b2a10012
