# Supplementary material for: Impact of peripheral artery disease on short-term outcomes after percutaneous coronary intervention: A report from Japanese nationwide registry
Source: PLoS One. 2020 Oct 6;15(10):e0240095. doi: 10.1371/journal.pone.0240095 (PMC7537874; doi:10.1371/journal.pone.0240095)
Supplement: Attachment — Submitted filename: PONE-D-20-00744_R1.pdf [file pone.0240095.s003.pdf]

PONE-D-20-00744 R1

Impact of peripheral artery disease on short-term outcomes after percutaneous coronary intervention: A report from Japanese nationwide registry

Although the revision has improved the manuscript, I still have some concerns:

- Reviewer 1 raised a concern regarding the treatment of continuous variables. I think this is still an important, unsolved point. On page 7 of the manuscript, the authors mention a large number of continuous variables (e.g. diastolic/systolic blood pressure, LDL-cholesterol level, triglyceride levels, . . .) that are all dichotomized into clinical fenotypes such as hypertension or dyslipidemia. If possible, I suggest that the authors use the continuous variables instead of the clinical fenotypes. I might be possible, for instance, that only LDL-cholesterol levels impact the mortality risk and not the triglyceride levels. This distinction is lost if you use the clincial fenotypes. This is a missed opportunity, especially since the dataset contains a large number of observations.

  If these continuous variables are not available, please mention this in the discussion section.
- The +- symbol is used in the abstract without explanation. Please remove this or mention what it means.
- Table 1: Remove the column with 'all patients'.
- Table 4: Round all odds ratios up to 2 or 3 decimals.
- The added sentences at the end of the limitations on page 18 should be removed. Even with only 1 continuous variable, it is possible to explore non-linear associations. In addition, what do the authors want to say with *Finally, even with the multivariate analysis, unmeasured and residual confounding could affect the results.*? I would remove this sentence.
