# Supplementary material for: Impact of peripheral artery disease on short-term outcomes after percutaneous coronary intervention: A report from Japanese nationwide registry
Source: PLoS One. 2020 Oct 6;15(10):e0240095. doi: 10.1371/journal.pone.0240095 (PMC7537874; doi:10.1371/journal.pone.0240095)
Supplement: Attachment — Submitted filename: Response to Reviewers.docx [file pone.0240095.s004.docx]

Responses to the Editor

>> We appreciate the time and expertise of the editors and reviewers and have revised our manuscript according to their suggestions.

*1. Reviewer 1 raised a concern regarding the treatment of continuous variables. I think this is still an important, unsolved point. On page 7 of the manuscript, the authors mention a large number of continuous variables (e.g. diastolic/systolic blood pressure, LDL-cholesterol level, triglyceride levels,. . .) that are all dichotomized into clinical fenotypes such as hypertension or dyslipidemia. If possible, I suggest that the authors use the continuous variables instead of the clinical fenotypes. I might be possible, for instance, that only LDL-cholesterol levels impact the mortality risk and not the triglyceride levels. This distinction is lost if you use the clincial fenotypes. This is a missed opportunity, especially since the dataset contains a large number of observations.*

*If these continuous variables are not available, please mention this in the discussion section.*

>> Thank you for your important suggestion. Unfortunately, the J-PCI registry does not include continuous variables such as diastolic/systolic blood pressure, LDL-cholesterol level, triglyceride levels, and so on (these variables were used by the local investigators/ data managers to define hypertension and dyslipidemia, but the numbers themselves were not recorded). As suggested by the editor, we mentioned this in the discussion section.

(Added sentence)

Discussion section (page R18, lines 10-13): Finally, since the J-PCI registry does not include continuous variables such as diastolic/systolic blood pressure, LDL-cholesterol level, triglyceride levels, and so on, we could not evaluate the impact of these continuous variables on the clinical outcomes.

*2. The +- symbol is used in the abstract without explanation. Please remove this or mention what it means.*

>> Thank you for pointing it out. The ± symbol indicated the standard deviation. We revised the sentence as follows:

(Before revision)

For the present study, we extracted data of 894,014 percutaneous coronary intervention (PCI) cases performed between 2014 and 2017 (mean age 70.2±11.0 years).

(After revision)

Abstract section (page R2, lines 9-11): For the present study, we extracted data of 894,014 percutaneous coronary intervention (PCI) cases performed between 2014 and 2017 (mean age 70.2 years [standard deviation 11.0]).

*3. Table 1: Remove the column with 'all patients'.*

>> Thank you for pointing it out. As suggested, we removed the column with `all patients`.

(Revised Table)

Results section (page R9-10): Table 1

*3. Table 4: Round all odds ratios up to 2 or 3 decimals.*

>> Thank you for pointing it out. As suggested, we rounded off all odds rations to three decimal places.

(Revised Table)

Results section (page R13-14): Table 4

*4. The added sentences at the end of the limitations on page 18 should be removed. Even with only 1 continuous variable, it is possible to explore non-linear associations. In addition, what do the authors want to say with Finally, even with the multivariate analysis, unmeasured and residual confounding could a ect the results? I would remove this sentence.*

>> Thank you for important suggestions. As suggested, we removed these sentences.

(Removed sentences)

Discussion section: Seventh, since all the variables except for age were categorical variables, it was not possible to explore non-linear relationships amongst the variables. Finally, even with the multivariate analysis, unmeasured and residual confounding could affect the results.
